# Comparative Genomic Analysis of Novel *Bifidobacterium longum* subsp. *longum* Strains Reveals Functional Divergence in the Human Gut Microbiota

**DOI:** 10.3390/microorganisms9091906

**Published:** 2021-09-08

**Authors:** Romina Díaz, Alexis Torres-Miranda, Guillermo Orellana, Daniel Garrido

**Affiliations:** Department of Chemical and Bioprocess Engineering, School of Engineering, Pontificia Universidad Catolica de Chile, Santiago 7820436, Chile; rgdiaz2@uc.cl (R.D.); aitorres2@uc.cl (A.T.-M.); gaorellana@uc.cl (G.O.)

**Keywords:** *Bifidobacterium longum* subsp. *longum*, comparative genomics, human gut microbiota

## Abstract

*Bifidobacterium longum* subsp. *longum* is a prevalent group in the human gut microbiome. Its persistence in the intestinal microbial community suggests a close host-microbe relationship according to age. The subspecies adaptations are related to metabolic capabilities and genomic and functional diversity. In this study, 154 genomes from public databases and four new Chilean isolates were genomically compared through an in silico approach to identify genomic divergence in genes associated with carbohydrate consumption and their possible adaptations to different human intestinal niches. The pangenome of the subspecies was open, which correlates with its remarkable ability to colonize several niches. The new genomes homogenously clustered within subspecies *longum*, as observed in phylogenetic analysis. *B. longum* SC664 was different at the sequence level but not in its functions. COG analysis revealed that carbohydrate use is variable among *longum* subspecies. Glycosyl hydrolases participating in human milk oligosaccharide use were found in certain infant and adult genomes. Predictive genomic analysis revealed that *B. longum* M12 contained an HMO cluster associated with the use of fucosylated HMOs but only endowed with a GH95, being able to grow in 2-fucosyllactose as the sole carbon source. This study identifies novel genomes with distinct adaptations to HMOs and highlights the plasticity of *B. longum* subsp. *longum* to colonize the human gut microbiota.

## 1. Introduction

*Bifidobacterium longum* species are among the first microbial colonizers of the human gastrointestinal tract. Commonly, the presence of these microorganisms is associated with positive temporary and long-term effects on host health [1,2,3,4]. As a result, certain *Bifidobacterium longum* strains are commonly used as probiotics in the food industry [5,6]. This species is composed of three subspecies: *B. longum* subsp. *infantis*, *B. longum* subsp. *longum* and *B. longum* subsp. *suis* [7,8,9]. Among the key phylogenetic groups of *Bifidobacterium*, *B. longum* subsp. *longum* appears to be widely distributed in both the infant and the adult gut microbiome [1,6], in contrast to certain species that appear to be exclusively found in infants (*B. longum* subsp. *infantis* and *Bifidobacterium breve*) or only in adults (*Bifidobacterium adolescentis*, *Bifidobacterium pseudocatenulatum*) [2,10].

Studies concerning the functional classification of the bifidobacterial pangenome have revealed that approximately 14% of annotated genes are involved in carbohydrate metabolism, focusing on polysaccharides such as inulin and arabinoxylan [4,11]. In this regard, some strains of *B. longum* subsp. *longum* have demonstrated a remarkable adaptation in HMO (human milk oligosaccharide) use, including enzymes that metabolize different types of HMO, releasing building blocks such as lacto-N-biose and galacto-N-biose [12,13,14]. Moreover, some strains can use fucosylated HMOs such as 2FL (2-fucosyllactose) or 3FL (3-fucosyllactose) [15,16,17]. In addition, proteomic analysis has shown the ability of a few isolates to break down the host-derived mucus glycans, which would be critical for *B. longum* subsp. *longum* adhesion to the intestinal epithelium and the establishment of its natural ecological niche [2,4,18,19].

Furthermore, *B. longum* subsp. *longum* participates in synergy with *Bacteroides* strains to degrade xylose- and mannose-containing carbohydrates, in addition to relies on pathways playing an important role in mucus use [20,21,22,23]. Therefore, *B. longum* subsp. *longum*’s characteristics, including the consumption of HMOs and other complex dietary carbohydrates along with their interactions with the host, have allowed its establishment, cross-feeding interactions, and co-evolution in both the infant and adult human gut microbiome.

Genomic and functional analyses have been crucial to unravel the genetic strategies adopted by the subspecies *longum*. These studies pinpoint competitive advantages to colonize the human intestinal tract, which appear to be facilitated by their adaptation to a glycan-rich environment [2,24,25]. In this context, the diverse glycosyl hydrolase (GH) gene repertoires reflected physiological traits that could partially explain the successful adaptation of *B. longum* subsp. *longum* to the different ecological niches, metabolizing a wide range of sugars and interacting with other commensal microorganisms. [7,26]. In addition, comparative genomic studies of *B. longum* subsp. *longum* have reflected an adaptation to their specific host through an evolutionary process involving the core genome, the accessory gene composition, and the specific gene set of the pangenome [26,27].

Thus, the integration of comparative and functional genomics reveals genetic diversity, critical genetic factors, and evolutionary adaptation of *B. longum* subsp. *longum* strains in the human gut microbiome at specific life stages [14,28].

Although we have advanced in our knowledge regarding the adaptation of *B. longum* subsp. *longum* in the human gastrointestinal tract, its genome plasticity, and genetic mechanisms used to metabolize a large group of carbohydrates in both the infant and adult gut microbiome makes them far from fully understood. Therefore, it is especially noteworthy to include in these analyses genomes with novel features. In this context, this study explores the genomic bases by which 154 selected genomes of *B. longum* persist in the different ecological niches of the human gut microbiome through a comparative analysis and describes their multiple capabilities to evolve with the host through an in-silico approach, including four new strains isolated from Chilean young adults.

## 2. Materials and Methods

### 2.1. Bacteria and DNA Extraction

Our study included a total of 154 *Bifidobacterium longum* genomes (including the subspecies *infantis*, *longum*, and *suis*), which were retrieved from the Department of Energy’s Joint Genome Institute Microbial Genomes and Microbiome (IMG) and the National Center for Biotechnology Information (NCBI). In addition, four *B. longum* subsp. *longum* Chilean strains (D4, M12, E1, and S3) previously isolated from young adult fecal samples were included [29]. *B. longum* Chilean strains were routinely grown on Man-Rogosa-Sharpe (MRS) broth (Difco Laboratories, Detroit, MI, USA) supplemented with 0.05% *w*/*v* of L-cysteine-HCl (Sigma-Aldrich, St. Louis, MO, USA). Cultures were incubated at 37 °C for 24–48 h in an anaerobic jar (Anaerocult, Merck, Darmstadt, Germany) with anaerobic packs (Gaspak EM, Becton-Dickinson, Franklin Lakes, NJ, USA).

Genomic DNA extraction was performed using a modified version of a phenol-chloroform isoamyl protocol [30]. Briefly, cell pellets were lysed with lysozyme incubated at 37 °C for 30 min (Amresco, Toronto, ON, Canada). The suspensions were purified using phenol:chloroform:isoamyl-alcohol 25:24:1 pH 8 and sterile acid-washed glass beads (Sigma-Aldrich, St. Louis, MO, USA). Cells were disrupted using a Disruptor Genie for 6 min (Scientific Industries, Bohemia, NY, USA) and centrifuged for phase separation. The obtained supernatants were purified with chloroform:isoamyl-alcohol 24:1 and centrifuged for phase separation. DNA was precipitated with isopropanol and sodium acetate 3M. Precipitated DNA was pelleted by centrifugation, washed with cold ethanol (biology molecular grade) twice, and dried for ethanol evaporation. DNA concentrations were calculated by measuring the absorbance at 260 nm using an Infinite 200 PRO spectrophotometer (Tecan Trading AG, Infinite M200 PRO, Männedorf, Switzerland) [31].

### 2.2. Genome Sequencing, Assembly, and Functional Prediction

Genomic DNA sequencing was performed at MicrobesNG (Birmingham, U.K.) on the Illumina MiSeq platform sequencing the v3 region with paired-end sequencing. Reads were trimmed using Trimmomatic [32]. Trimmed reads were de novo assembled following a pipeline incorporating Mira, MaSuRCa, and SPAdes software with default parameters [33,34,35]. The results were merged with the SPAdes untrusted-contigs option. Prediction of putative open reading frames (ORFs) was performed using Prodigal in all contigs from assembly genomes [36]. Identified ORFs were then automatically annotated based on BLASTp analysis [37], refined, and verified using the multiple software annotations (EggNOG-MAPPER and InterPro) [38,39], in addition to the protein family Pfam database [40].

Glycosyl hydrolases in the genome of *B. longum* subsp. *longum* were predicted and annotated using the Carbohydrate-Active Enzymes (CAZy) database via the Database for Automated Carbohydrate-Active Enzyme Annotation (dbCAN) meta server [41,42]. The CAZy hits were achieved considering at least two of the three annotations tools (HMMER v.3.2, DIAMOND v.0.9, and Hotpep v.1). Enzyme commission number (EC number) was annotated using ECPred v.1.1 with default parameters [43]. Glycosyl hydrolases (GHs) profiles were visualized with a heatmap generated by R with ggplot2 package [44]. GHs profiles (rows) were clustered using the “average” method (UPGMA) implemented in the R *hclust* function.

### 2.3. Pangenome and ANI Analysis

Pangenome analysis was performed considering uniquely the subspecies *longum* to avoid functional divergence when studying the three *B. longum* subspecies. The *B. longum* subsp. *longum* genomes were retrieved from the IMG and NCBI public databases (at the moment to begin this study), and four *B. longum* Chilean isolates (D4, M12, E1, and S3) were added to the analysis. Initially, each assembly was annotated with PROKKA v1.14.15 using default parameters [45]. Next, the pangenome’s size, core genome, and unique genes were established with Roary v.3.12.0 using PROKKA gff outputs as inputs [46]. Roary was used with a minimum of 95% of identity. The Roary pangenome statistics summary output was visualized using the ggplot2 package from R [47]. Pangenome sequences were provided in the pangenome_reference.fa output file in Roary. These sequences were inputted into the EggNOG 4.5.1 eggNOG-mapper v2 genome-wide functional annotation online tool [38]. The results were downloaded and organized based on clusters orthologous groups (COGs) found in *B. longum* Chilean isolates and *B. longum* subsp. *longum* publicly available. Values associated with COG categories represent the percentage of COGs belonging to each category out of the total number of identified COGs. If a gene was assigned to two COG categories, each COG category was counted separately.

The average nucleotide identity (ANI) among *B. longum* subsp. *longum* genomes were calculated with pyani v.0.2.8 using the ANIm MuMmer method [48] and plotted with python and seaborn packages.

### 2.4. Phylogenetic Inference of B. longum subsp. longum Genomes

Phylogenetic inference of 154 *B. longum* subsp. *longum* obtained from public databases, and the four Chilean isolates were achieved by aligning the core set pangenome genes. In addition, *Bifidobacterium breve* UCC2003 was used as an outgroup and obtained from the IMG database. Each set of orthologous proteins was aligned using MAFFT v7. The L-INS-i option was concatenated into a large alignment for each genome [49]. Moreover, the pipeline used OrthoFinder v.2.4.1 and RaxML v.8.1.24 for the likelihood of tree construction [50,51]. OrthoFinder computed each set of orthologous proteins with default parameters. RaxML was executed with the PROTGAMMAAUTO option for amino acid substitution and MRE stop-bootstrapping criteria [52]. Finally, the phylogenetic tree was visualized and formatted using the ggplot2 and ggtree R packages [53].

### 2.5. Gene Cluster Analysis

A local BLASTp with an E value of 1 × 10^−5^, assuming 60% or above sequence identity and coverage, was performed to obtain the shared genes among the total genomes. Protein sequences previously described were used for a specific HMO cluster in *B. longum* M12 [15]. BLASTp output was parsed using in-house Perl, python, and bash scripts. Cluster search results were visualized with gene arrow maps generated by R language using the gggenes package [54].

### 2.6. Detection of Virulence Factor and Antibiotic Resistance Genes

VFDB database amino acid sequences [55,56] were used to evaluate the virulence potential of *B. longum* Chilean isolates and each *B. longum* subsp. *longum* strain using BLAST. BLASTp was performed for sequence similarity search; the output was parsed using in-house Perl and bash scripts. Sequences matched with E values of 1 × 10^−5^, sequence identity, and coverage of 60% or above were considered homologs. Moreover, the Comprehensive Antibiotic Resistance Database (CARD; http://card.mcmaster.ca; accessed on 2 November 2020) [57] was used to detect bacterial antimicrobial resistance. The Resistance Gene Identifier (RGI) software was used for resistome analysis and prediction in *B. longum* subsp. *longum* sequences. Heatmaps of VFDB and CARD were generated using R language and ggplot2 package.

### 2.7. HMO Growth Conditions

Two out of four Chilean isolates *(B. longum* M12 and *B. longum* D4) were evaluated for their ability to use human milk oligosaccharides as a sole carbon source. Briefly, the strains cultures were grown for 24 h at 37 °C on Man-Rogosa-Sharpe (MRS) broth (Difco Laboratories, Detroit, MI, USA) supplemented with 0.05% *w*/*v* of L-cysteine-HCl (Sigma-Aldrich, St. Louis, MO, USA). Afterward, the cultures were transferred on a modified ZMB medium (mZMB) for 24 h at 37 °C [58]. Ten μL of each overnight culture was used to inoculate 200 μL of mZMB, supplemented with 2% (*w*/*v*) lactose, 1% (*w*/*v*) 2-fucosyllactose (2FL), 1% (*w*/*v*) Lacto-N-tetraose (LNT), or 1% (*w*/*v*) Lacto-N-neotetraose (LNnT) respectively. For all cases, the cultures in the microplates were covered with 20 μL of sterile mineral oil to avoid evaporation. The incubations were carried out at 37 °C for 48 h in anaerobic conditions (Sheldon Manufacturing INC, Bactronez-2 Anaerobic Chamber Workstation, Cornelius, OR, USA). Cell growth was monitored in real-time by assessing optical density (OD) at 620 nm using a Tecan F50 Microplate Spectrophotometer (Tecan Trading AG, Infinite F50, Männedorf, Switzerland) every 30 min preceded by 5 s shaking at variable speed. The OD620 values were plotted using R and ggplot2.

### 2.8. Plasmid Prediction and Mobile Genetics Elements

Plasmid prediction was performed using plasmidSpades [59] with default parameters. Spades predicts plasmid contigs using a coverage filter approach for the assembly graph. However, plasmid prediction from whole-genome sequencing short reads is an unresolved problem, so we used Platon [60] (with default parameters in *accuracy* mode) as a comparison point to the plasmidSpades results. Platon uses a pre-computed database with a set of protein-coding genes and their associated replicon distribution score (RDS), which is used to distinguish between plasmid or chromosome-related contigs from draft assemblies. Plasmid predicted contigs were compared against PLSDB [61] database v.2020_11_09 (available in https://ccb-microbe.cs.uni-saarland.de/plsdb/; accessed on 2 August 2021), a database constructed with an updated collection of plasmid records from NCBI nucleotide database, using MASH [62] distance estimation software with the *screen* option. Finally, Chilean isolates mobile genetics elements were annotated with MobileElementFinder [63].

## 3. Results

### 3.1. Bifidobacterium longum subsp. longum General Features

In order to determine the general genomic characteristics of four *B. longum* strains previously isolated from Chilean subjects (D4, M12, E1, and S3), their genomes were sequenced and subsequently de novo assembled. The average genome size of *B. longum* isolates was 2.35 Mb with a minimum of 2.27 Mb in *B. longum* S3 and a maximum of 2.49 Mb in *B. longum* E1 (Supplementary Table S1). The GC content ranged between 59.70% (*B. longum* M12) and 60.33% (*B. longum* E1). These genomic characteristics are consistent with those previously reported for other *Bifidobacterium* genomes [1,26].

**Figure 1:**
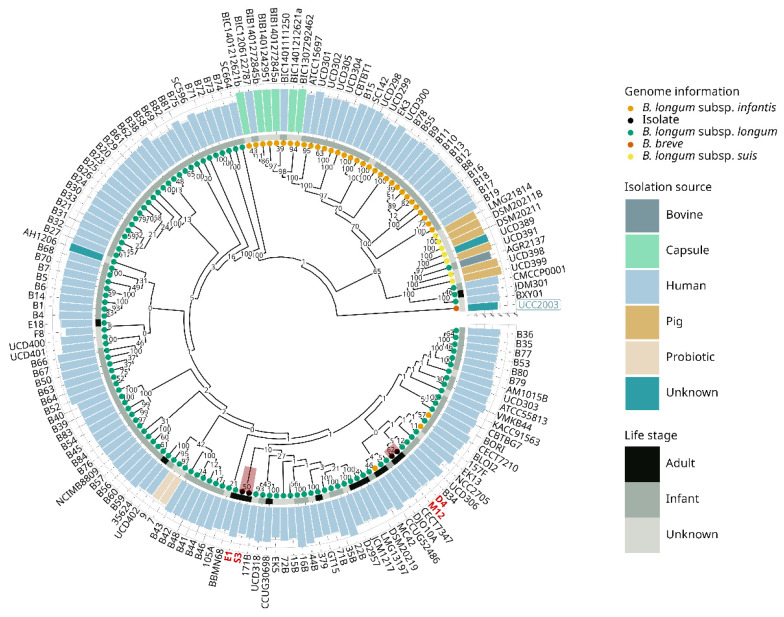
Phylogenetic tree of 158 *B. longum* subsp. *longum* strains showing metadata and bootstrap value between genomes. Colored bars and dots indicate genome information, isolation source, and life stage. Chilean strains are colored in red. *Bifidobacterium breve* UCC2003 was used as an outgroup.

Considering *B. longum* subsp. *longum* genomes available in public databases (IMG and NCBI) at the beginning of the study and the Chilean isolates, the average number of total genes did not present significant differences among the total of selected genomes via unpaired *t*-test (*p* < 0.05). In addition to the phylogeny analysis (Figure 1), the 16S rRNA gene sequence of each *B. longum* Chilean isolate was compared with the 16S rRNA sequence database employing BLASTn to denominate the *Bifidobacterium* subspecies. Thus, the four novel strains were assigned by homology to subspecies *longum* taxonomic group (Supplementary Table S1).

### 3.2. Evolutive Phylogenetic Inference

A phylogenetic tree was constructed employing the protein sequences from the core genomes, which are considered conserved molecular markers to analyze the evolutive relationship of four Chilean strains with representatives *B. longum* genomes (subspecies *infantis*, *longum*, and *suis*) (Figure 1). The phylogenetic tree clustered the genomes following their previous taxonomic organization, reflecting divergence among the branches in the subspecies that share a common ancestor. As expected, we observed that most genomes were positioned uniformly segregating into subspecies *longum* taxonomic groups. Excluding the outgroup *B. breve* UCC2003, the entire phylogenetic tree was divided into two principal clades with high support values of 100 for *B. longum* subsp. *longum*, and 65 for *B. longum* subsp. *infantis* and *suis*. This last branch was divided into two principal clades with high support values of 100, resulting in one clade for the *B. longum* subsp. *infantis* and one clade for the *B. longum* subsp. *suis*. Furthermore, low support values were observed among branches in the *B. longum* subsp. *longum* clade due to the high similarity of core genes among the different strains of *B. longum* subsp. *longum* analyzed at the protein sequence level.

Most infant gut isolates clustered together in the same branch, and adult gut *B. longum* subsp. *longum* genomes appeared interspersed across infant genomes (Figure 1). Even if the *B. longum* SC664 genome was clustered in the subspecies *longum* clade, it appears very divergent in comparison with other genomes (Figure 1).

We found specific conflicts with particular genomes that were previously assigned to different subspecies clades. For example, *B. longum* subsp. *longum* AGR2137 was assigned to the subspecies *suis* branch, while *B. longum* subsp. *infantis* 157F, CECT7210, and CCUG52486 were categorized in the subspecies *longum* taxonomic group. Although some studies have corroborated the correct annotation, these misidentifications in *Bifidobacterium longum* subspecies represent a permanent challenge in the distinct subspecies identification because of the close relationship between the subspecies [12,64].

Regarding the novel isolates, the phylogenetic tree arrangement revealed that *B. longum* D4 and *B. longum* M12 clustered in the same node with a bootstrap support value of 62. Moreover, identical categorization was observed in *B. longum* E1 and *B. longum* S3, which obtained a bootstrap support value of 50 considering 345 replicates in the inference analysis. The four Chilean strains were homogeneously distributed in the branch constituting members of the subspecies *longum* taxonomic group (Figure 1). Nonetheless, *B. longum* D4 and *B. longum* M12 could be genetically different from *B. longum* E1 and *B. longum* S3, predominantly for their subclade position into the tree.

### 3.3. Predictive Genomics Analysis

#### 3.3.1. Average Nucleotide Identity (ANI) Analysis

**Figure 2:**
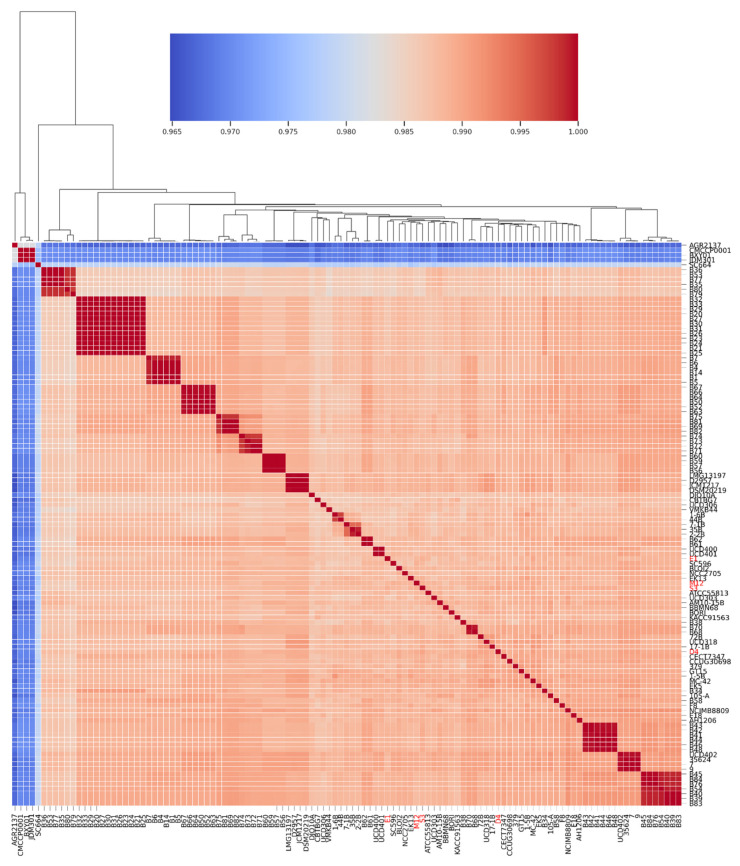
Heatmap representing the percentage of average nucleotide identity (ANI) of 158 *B. longum* subsp. *longum* strains. The color key represents the percentage identity among strains with lower (blue) and higher (red) ANI values. The strains are clustered in dendrograms based on row means. The Chilean strains are colored in red.

In order to define the genomic relationship among *B. longum* subsp. *longum* genomes (from public databases and the novel strains), an average nucleotide identity (ANI) analysis was performed with all genomes selected for this study. The genomes were largely clustered into an ANI arrangement reaching values of over 0.985 (Figure 2). Interestingly, some genomes obtained an ANI range below 0.97. For instance, *B. longum* AGR2137, isolated from calf feces, had the lowest ANI value, which elucidates the genomic divergence between strains inhabiting the animal or the human gut microbiome. This proportion was similar to the ANI range of *B. longum* CMCC P0001, BXY01, and JDM301. Regarding *B. longum* SC664, the ANI value was approximately 0.98. In this case, we observed a significant separation in the heatmap, suggesting that *B. longum* SC664, isolated from the infant gut microbiome, presents genome-wide variations supported by the phylogenetic inference (Figure 1).

Most *B. longum* subsp. *longum* genomes had ANI values >0.99. However, they showed enough differences that allowed their discrimination in the ANI matrix, similar to Figure 1. Regarding *B. longum* genomes that obtained an ANI value close to 1.000, we can presume that these strains were isolated from the same source. For example, *B. longum* B50, B52, B63, B64, B66, and B67 were isolated from a formula-fed infant during the first 18 months of life [65]. Similarly, *B. longum* B35, B36, B53, B70, B77, and B80 were isolated from the same breastfed infant, which would support the ANI arrangement. According to the Chilean strains, we observed a homogeneous distribution and an ANI range close to 0.99, corroborating the subspecies categorization and the divergence with genomes such as *B. longum* SC664 (Figure 2).

#### 3.3.2. *Bifidobacterium longum* subsp. *longum* Pangenome

**Figure 3:**
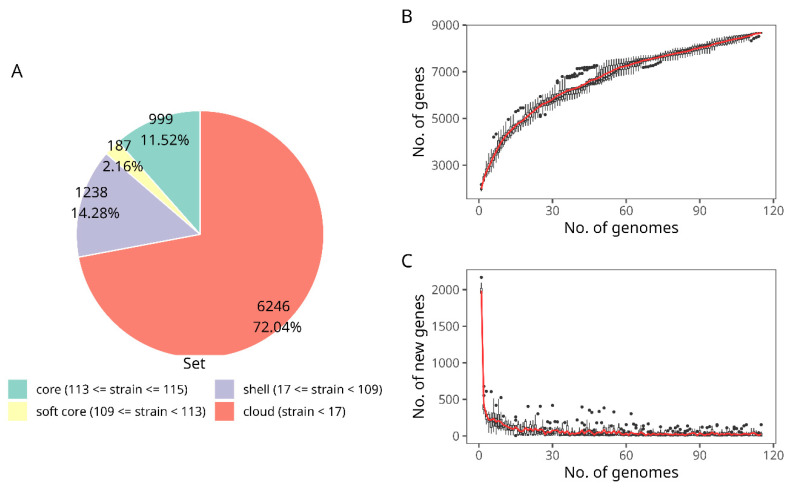
Pangenome of *B. longum* subsp. *longum* strains. (**A**) Pie chart indicating the gene set in the pangenome. (**B**) Plot of the accumulate number of new genes against the number of genomes added in the pangenome. (**C**) Number of genes attributed to the core genome versus the number of genomes added in the core genome plot.

The genome of novel *B. longum* strains and 115 *B. longum* genomes from public databases were considered to visualize the pangenome. This analysis was made with 8670 cluster genes found in 115 selected genomes (Figure 3A). It revealed that the pangenome comprises 999 core genes, 187 softcore genes, 1238 shell genes, and 6246 cloud genes.

The pangenome frequency showed a proportional relationship between the number of genes and genomes. Accordingly, while more genomes were proposed, the number of new genes was lower. Moreover, we found that after the addition of the 115th genome, more strains could be necessary to describe any increment in the pangenome curve (Figure 3B). Consistent with the above, the core genome revealed a steady and asymptotic trend after adding the 115th genome at approximately 150 genes (Figure 3C). Consequently, considering both the curve analysis and the current accessibility of *B. longum* subsp. *longum* genomes, we suggest that the pangenome is not entirely closed but approaching this state.

**Figure 4:**
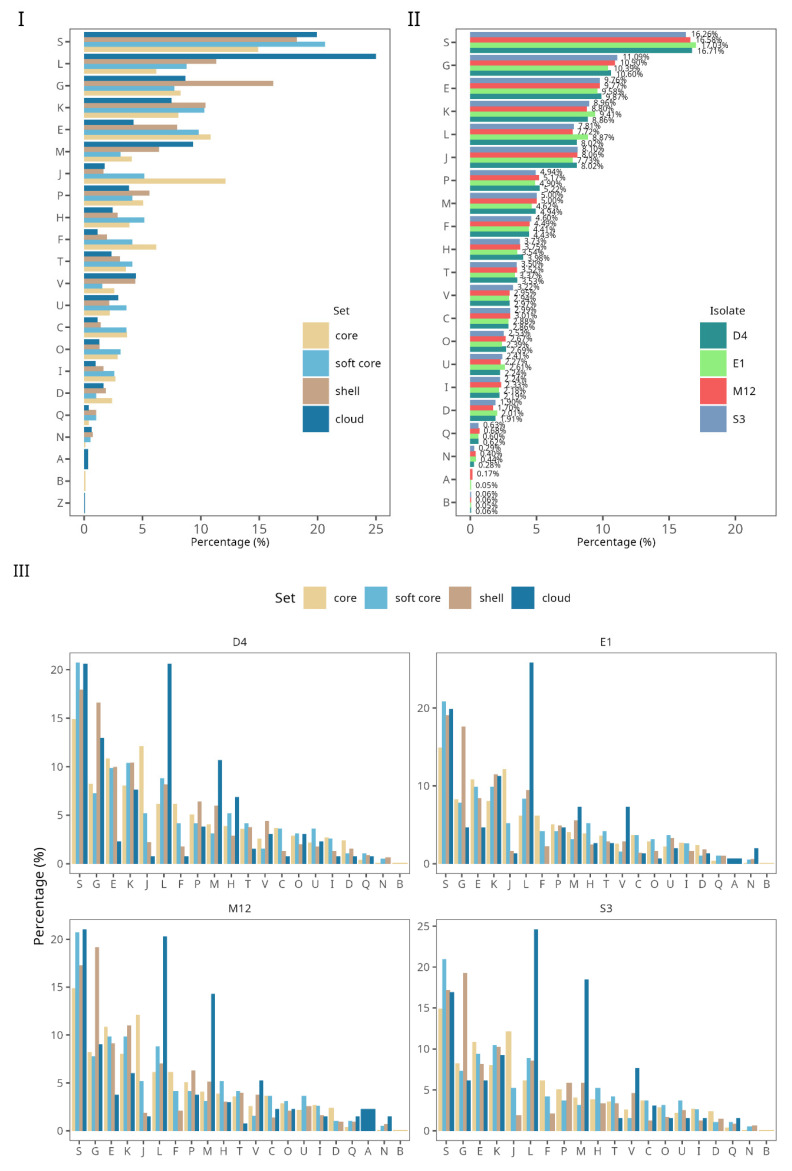
Percentage of total cluster orthologous group (COGs) annotated in *B. longum* subsp. *longum* strains. (**I**) Distribution of COG functional categories in each pangenome gene set. (**II**) Percent of each GOG functional category in *B. longum* subsp. *longum* Chilean strains. (**III**). Distribution of COG functional categories in *B. longum* subsp. *longum* Chilean strains. The COGs categories are RNA processing and modification (A), chromatin structure and dynamics (B), energy production and conversion (C), cell cycle control, cell division, chromosome partitioning (D), amino acid transport and metabolism (E), nucleotide transport and metabolism (F), carbohydrate transport and metabolism (G), coenzyme transport and metabolism (H), lipid transport and metabolism (I), translation, ribosomal structure and biogenesis (J), transcription (K), replication, recombination and repair (L), cell wall/membrane/envelope biogenesis (M), cell motility (N), post-translational modification, protein turnover, and chaperones (O), inorganic ion transport and metabolism (P), secondary metabolites biosynthesis, transport and catabolism (Q), function unknown (S), signal transduction mechanisms (T), intracellular trafficking, secretion, and vesicular transport (U), defense mechanisms (V), and cytoskeleton (Z).

In addition, we evaluated the cluster orthologous groups (COGs) according to their occurrence in each gene set that makes up the pangenome (Figure 4). The COGs functional categories showed a variable distribution among those four-gene sets in the pangenome. The “replication, recombination and repair (L)” category associated with genetic transference presented a higher percentage in the cloud gene set. Moreover, in the shell gene set, “replication, recombination, and repair (L)” and “carbohydrate transport and metabolism (G)” were the most representative functional categories, indicating that these are more genetically variable processes across *B. longum* strains. Finally, “RNA processing and modification (A)”, “cytoskeleton (Z)”, and “chromatin structures and dynamics (B)” were found exclusively in the cloud and core gene set, respectively, indicating conserved functions (Figure 4I). “Translation, ribosomal structure and biogenesis (J)” (~13%), and “amino acid transport and metabolism (E)” (~11%) were the categories with the highest percentages observed in the core gene set. In summary, these results indicate a large degree of conservation in the functions of *B. longum* subsp. *longum* genomes (Figure 4I).

Considering the COG categories investigated in the *B. longum* Chilean strains, we found that the highest percentages were distributed in the categories belonging to “function unknown (S)” (16.65%), “carbohydrate transport and metabolism (G)” (10.74%), and “amino acid transport and metabolism (E)” (9.74%) (Figure 4II). In addition, all the functional categories were homogeneously distributed in each isolated strain, which could suggest a lower diversity among the Chilean isolates (Figure 4II).

Moreover, we studied the COG categories distribution in each isolated Chilean strain according to the number of genes. Thereby, *B. longum* D4, M12, E1, and S3 obtained a similar number of genes in the “function unknown (S)”, “carbohydrate transport and metabolism (G)”, and “amino acid transport and metabolism (E)”, all of them represented in the core gene set (Figure 4III). Furthermore, The COGs distributions were similar between the Chilean strains and *B. longum* subsp. *longum* genomes used in this study.

#### 3.3.3. Glycosyl Hydrolase Prediction

**Figure 5:**
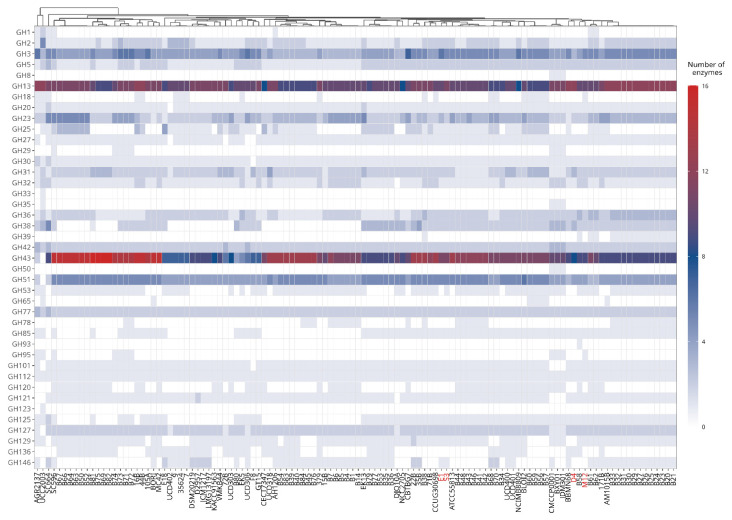
Heatmap displaying the predicted glycosyl hydrolases family members identified in *B. longum* subsp. *longum* genomes. Chilean strains are colored in red.

The *Bifidobacterium* genus is recognized for its specialization in the fermentation of a wide variety of complex carbohydrates [17,25,26]. Thereby, we considered representatives *B. longum* subsp. *longum* from public databases and Chilean isolates strains to evaluate and define the distribution of glycosyl hydrolase (GH) genes in their genomes using the CAZy database. This predictive analysis revealed the presence of 39 GHs families distributed among selected *B. longum* subsp. *longum* strains (Supplementary Table S2). Glycosyl hydrolases belonging to family 13 (GH13, α-glucosidases) and family 43 (GH43, including α-arabinofuranosidases and β-xylosidases) were predominant in all *B. longum* subsp. *longum* genomes (Figure 5). The distribution of other GHs was not uniform among all strains. For instance, GH3 (β-glucosidase) and GH51 (α-L-arabinofuranosidase) were present between two and six enzymes for each genome, while GH2 and GH42 (β-galactosidases), GH5, GH31, GH32, GH36, and GH127 were found between one and two copies in each *B. longum* subsp. *longum* genome. In addition, a β-glucosidase (GH1; EC 3.2.1.21), which is associated with the hydrolysis of numerous glycosides and oligosaccharides, was found in strains such as *B. longum* SC664, *B. longum* SC596, and *B. longum* AGR2137. This GH is interesting because could suggest the adaptation of some *B. longum* strains to the different human intestinal niche, supposing the adaptations of these strains mainly in the human gut microbiome.

We also found the presence of GHs associated with the degradation and use of O-glycans such as glucosylceramidase (GH30; EC 3.2.1.45), which was conserved across all genomes. An endo-β-N-acetylglucosaminidase (GH85; EC 3.2.1.96) was found in nearly 70% of genomes, while an endo-α-N-acetylgalactosaminidase (GH101) and a β-L-arabinofuranosidase (GH121; EC 3.2.1.-) were found in mostly all *B. longum* subsp. *longum* genomes, including all Chilean strains.

A lacto-N-biosidase (GH136; EC 3.2.1.140) was found in a Chilean isolate (*B. longum* D4). GH136 hydrolyzes lacto-N-tetraose, one of the most abundant human milk oligosaccharides. In addition, the gene was detected in *B. longum* UCD306, *B. longum* BLOI2, *B. longum* AH1206, and *B. longum* MC-42, among other genomes (Figure 5).

Our analysis revealed that the *B. longum* M12 genome contains an α-1-2-L-fucosidase (GH95) associated with HMOs fucosylated consumption. Interestingly, a GH95 was also detected in *B. longum* BCY01, *B. longum* JDM301, and *B. longum* SC596, suggesting a possible niche adaptation of these strains in the infant gut microbiome. Regarding HMO use, α1-3/4-L-fucosidases (GH29) were rarely found in some *B. longum* subsp. *longum* genomes. In this regard, *B. longum* CMCCP0001, JDM301, BXY01, and SC596 were unusual in that they contained both GH29 and GH95 since these enzymes have been identified mainly in *Bifidobacterium* spp. colonizing the infant gut microbiome such as *B. longum* subsp. *infantis* and *Bifidobacterium bifidum* genomes [15].

In addition, we evaluated the presence of virulence factors and antibiotic resistance genes in *B. longum* genomes selected in this study. In this context, the VFDB database did not retrieve any virulence factor or pathogenic characteristics associated with *B. longum* Chilean strains (Supplementary Table S2, Supplementary Figure S1). The CARD database showed a limited distribution of genes associated with antibiotic resistance in *B. longum*. A small number of genomes contained genes providing resistance to vancomycin (3) or erythromycin (4), including *B. longum* E1 (Supplementary Table S2, Supplementary Figure S2).

#### 3.3.4. Complex Carbohydrates Use Cluster

**Figure 6:**
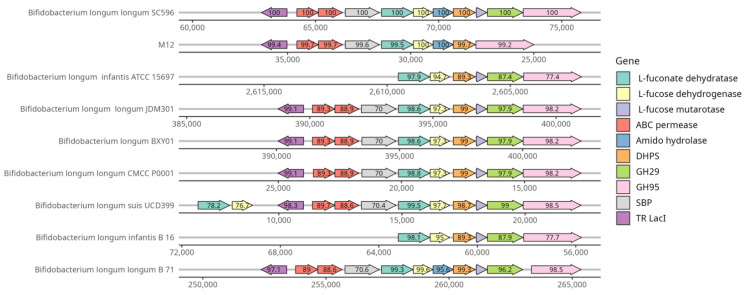
Locus map of carbohydrate use cluster identified in *B. longum* M12, and homologous *B. longum* subsp. *longum* genomes from public databases.

**Figure 7:**
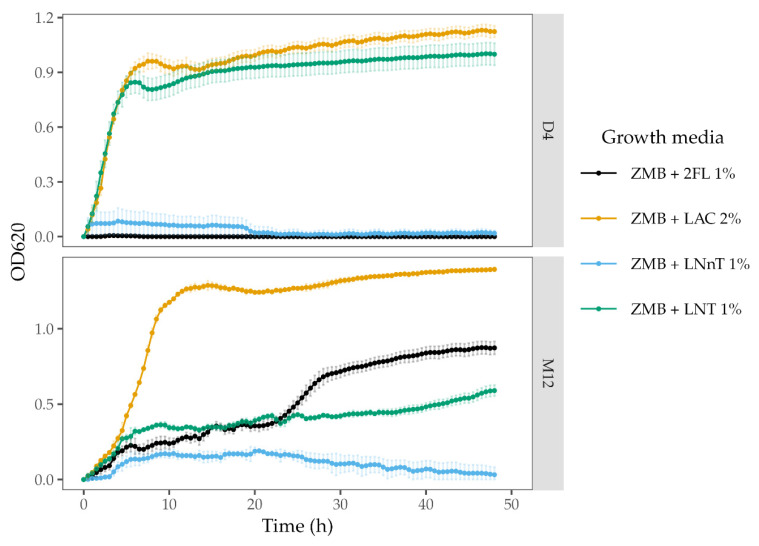
Growth curves of *B. longum* subsp. *longum* M12 and D4 inferred at 620 nm. The HMOs 2FL (2-Fucosyllactose), LNT (Lacto-N-tetraose), and LNnT (Lacto-N-neotetraose) were used as the only carbon source. Lac (Lactose) was used as a positive control.

The genomic analysis identified a genetic region in *B. longum* M12 similar to the FHMO (fucosylated human milk oligosaccharides use cluster) previously described in *B. longum* SC596 strain [15]. In silico analysis also revealed the cluster in 7 others out of 115 *Bifidobacterium longum* subsp. *longum* strains considered in this study (Figure 6). *B. longum* strains contained the genes encoding for the cluster transcriptional regulator (TR LacI), ABC transporters, fucose-metabolism enzymes, and at least one GH95 (α-1-2-L-fucosidase; EC 3.2.1.51). Interestingly, the *B. longum* M12 cluster did not contain any glycosyl hydrolase family 29 (GH29), as well as it appears to have lost the gene encoding for L-fucose mutarotase. These results suggest this strain has a limited fucose metabolism, only consuming α1-2-fucosyl-containing oligosaccharides such as 2-fucosyl lactose (2FL), but not α1-3/4-containing oligosaccharides. As expected, *B. longum* M12 was able to grow using 2FL and LNT as the sole carbon source, but not LNnT (Figure 7).

Regarding *B. longum* D4, we found a putative GH136 described previously in a *Bifidobacterium longum* [14]. GH136 is a lacto-N-biosidase that promotes bifidobacterial growth through neutral HMO consumption. Even though GH136 is predominantly found in *B. bifidum* genomes, there are certain *B. longum* strains capable of using the GNB/LNB pathway to consume LNT, releasing lacto-N-biose (LNB) and lactose (Lac) to the media [66]. Nevertheless, we did not identify a defined cluster in *B. longum* D4 comparable with the *B. bifidum* genome. The experimental in vitro assay demonstrated a vigorous growth of *B. longum* D4 in LNT as the only carbon source. However, it was not able to grow in other HMOs such as 2FL and LNnT (Figure 7).

#### 3.3.5. Plasmids and Mobile Elements

plasmidsSpades predicted the existence of plasmid contigs in *B. longum* D4, E1, and M12 strains (Supplementary Table S3); meanwhile, Platon identified plasmid-related contigs in *B. longum* D4 and *B. longum* E1 draft assemblies. Interestingly, plasmidSpades predicted a set of large contigs in the *B. longum* E1 isolate, two of them larger than 100 kb. In addition, there are contigs predicted by both approaches, as is the case for nodes 1 (plasmidSpades) and 14 (Platon) in the plasmid assemblies of *B. longum* D4 isolate (Supplementary Table S4).

The PLSDB search shows that the plasmids assembled for *B. longum* D4 and *B. longum* E1 isolates have overlap with other circular plasmids reported in *Bifidobacterium longum*, particularly in the subspecies *longum* and *infantis* (Supplementary Table S3). However, the best hits from each search correspond to small-sized plasmids that include mostly hypothetical proteins.

Finally, mobile genetics elements analysis detect 6 types of insertion sequences in Chilean isolates with a perfect or almost perfect hit (99–100% of sequence identity and coverage) (Supplementary Table S5).

## 4. Discussion

Comparative genomics studies of *B. longum* strains can provide insights into how different taxonomic groups adapt to the environment and what types of attributes are essential for these adaptations, whether related to the host or geographical environments [49]. Previous results obtained from the pangenome analysis have revealed that *B. longum* and *B. adolescentis* taxa show a higher genomic diversity than other bifidobacterial taxa such as *B. breve* and *B. bifidum* [27,67,68].

A closed pangenome has been defined as a finished pangenome in which there is no change when new genomes are added, and an open pangenome is defined as a pangenome that increases when a new genome is added [69]. It has been suggested that the open or closed nature of a pangenome is bound to the lifestyle of the studied bacterial species [70]. Under this context, the open pangenome is typical in species that colonize multiple environments and have multiple ways of exchanging genetic material. Some examples are *Streptococci*, *Meningococci*, *Helicobacter pylori*, *Salmonellae*, and *Escherichia coli* pangenomes. On the other hand, the closed pangenome bacteria are more conserved and live in isolated niches with limited access to the global microbial gene pool, i.e., with a low capacity to acquire foreign genes. Some examples are *Bacillus anthracis*, *Mycobacterium tuberculosis*, and *Chlamydia trachomatis* pangenomes.

*Bifidobacterium longum* subsp. *longum* could be found in different environments such as the oral cavity, the stomach, the large and small intestine of the human intestinal tract. In humans, it is dominant in the infant gut and commonly found in the adult gut microbiota, a property not commonly found among gut microbes. This particular dominance could partially explain why *B. longum* has access to exchanging genetic material with strains from different parts of the body, and therefore a common open pangenome. In this context, our study establishes that the pangenome is not entirely closed because more *B. longum* subsp. *longum* genomes are necessary to reach saturation. However, according to previous research [27], we could confer a subspecies-specific adaptation considering the core genome analysis, which is considered a conserved region genetic.

Regarding the Chilean strains used in this study, the observed COGs in each isolated strain were revealed as one of the higher percentages attributed to the “carbohydrate transport and metabolism (G)” functional category in the shell gene set. As a result, this could explain the higher diversity of the *B. longum* taxonomic group to consume a wide carbohydrate range compared with other *Bifidobacterium* taxa. In this regard, it has been described that 74% of secreted proteins are distributed among functions related to the “cell wall/membrane/envelope biogenesis (M)” and “carbohydrate transport and metabolism (G)” in bifidobacterial species [71]. These functions exert a crucial role in modulating the interaction with the host and the environment to acquire nutrients and therefore to establish the ecological niche [4,71]. In addition, the genomic comparison with *B. longum* subsp. *longum* genomes shows 581 gene families that are unique in the subspecies at taxonomic level, where 68% are associated with hypothetical functions, which reveals a high genomic diversity than *B. breve* taxa, while the remaining 32% is encoding precisely to mobile elements, ABC transporters, and glycosyl hydrolases, revealing the possible adaptations to specific substrates [67,72].

Remarkably, several observations are obtained from the *B. longum* SC664 phylogenetic organization. Our phylogenetic tree and the original annotation categorized the *B. longum* SC664 strain into the subspecies *longum* taxonomic group. *B. longum* SC664 was isolated from the infant gut microbiome, displaying a vigorous growth in neutral HMOs LNT (Lacto-N-tetraose) and LNnT (Lacto-N-neotetraose) [15]. In addition, *B. longum* SC664 possesses a gene (GH5; BLNM_00662) associated with cellobiose catabolism, which is detected in some genomes of *B. longum* subsp. *infantis* and *B. longum* subsp. *longum* AH1206 (data not shown), which indicates a possible adaptation due to trophic interactions of *B. longum* SC664 with other commensal microorganisms in the transition from the infant gut microbiome to the adult gut microbiome. *B. longum* SC664 could represent a niche adaptation but not necessarily a product of horizontal gene transfer, according to previous studies [8,25]. In addition, the ANI arrangement noticeably clustered the *B. longum* SC664 further away from Chilean isolated strains with a value below 0.98, which is closer to genomes such as *B. longum* AGR2137, previously isolated from the calf gut microbiome [27]. Moreover, the ANI analysis of Albert et al. clustered the *B. longum* SC664 genome conforming to subspecies *infantis* [8]. Notwithstanding, due to the large majority of *B. longum* genomes belonging to the subspecies *longum*, it is possible that fully sequenced of some strains such as *B. longum* SC664 and *B. longum* AH1206 indicated a different genomic architecture to adapt in their respective ecological niche [9].

We observed that *B. longum* M12 was a Chilean strain lacking the GH29 (α-1,3/4 fucosidase) carbohydrate enzyme family. A similar result has been reported in some *B. breve* (BR-07, BR-19, BR-C29, BR-H29, and BR-L29) and *B. pseudocatelatum* (CA-C29, CA-K29a, and CA-K29b) strains. These strains have grown in fucosylated HMO, only containing the GH95 family in their genomes [73]. Moreover, a phylogenetic analysis of the HMO cluster glycosyl hydrolase in *B. longum* strains (*infantis* and *longum* taxonomic group) has been investigated to determine the divergence between GH29 and GH95 [8]. The study of Albert et al. reported possible divergences in HMO use attributed to GH29 and GH95 nonsynonymous mutation.

Regarding *B. longum* D4, we observed one GH136 carbohydrate enzyme in its genome. A similar enzyme has been reported in a previous study of *B. longum* strains [14]. The conclusions obtained in the aforementioned study reflect that the consumption of type-1 HMOs (LNT) by some *B. bifidum* and *B. longum* strains can exert selective pressure and support the evolution of the symbiosis in the infant gut microbiome mediated by GH136 [14]. In addition, Asakuma et al. reported a pathway (GNB/LNB) by which *B. longum* subsp. *longum* JMC1217 intake LNB through the previous action of extracellular lacto-N-biosidase to degrade LNT [74]. These metabolic capabilities of *B. longum* play a vital role in the trophic interactions with other commensal bacterial communities, providing a mutualistic ecosystem in their host and allowing the cross-feeding interactions among microbes and the correct establishment of the gut microbiome.

## 5. Conclusions

*B. longum* subsp. *longum* is a subspecies with a higher genetic diversity than other *Bifidobacterium* taxa. This study reveals a genetic divergence between the four novel Chilean strains and representative *B. longum* genomes publicly available. In this regard, *B. longum* M12 and *B. longum* D4 isolated from Chilean young adults were able to consume fucosylated and neutral HMOs, respectively. These phenotypical characteristics indicated possible adaptation of Chilean strains to the human gut microbiome at different life stages. In addition, it is possible that *B. longum* D4 used a similar *B. bifidum* pathway to persist in the infant gut microbiota, which is interesting to evaluate the therapeutic capabilities of *B. longum* D4 in the infant gut microbiome.

The in silico and in vitro approaches performed in this work could explain the genetic divergence among some strains to identify the different strategies to adapt to the human gut microbiome. In addition, the evaluation of newly isolated genomes could contribute to the understanding of the specific adaptations of *B. longum* subsp. *longum* in the different ecological niches considering isolation sources and geographical conditions.

## Data Availability

Genome sequences are available with accession number PRJNA742412 and SRA codes SRR14996472, SRR14996473, SRR14996474, and SRR14996475.

## Supplementary Materials

The following are available online at https://www.mdpi.com/article/10.3390/microorganisms9091906/s1, Figure S1: Virulence factors heatmap, Figure S2: Antibiotic resistance heatmap, Table S1: Genomes features from public databases and Chilean isolates, Table S2: Virulence factors and antibiotic resistance information, Table S3: Plasmid and mobile elements in Chilean isolates, Table S4: Blast results of plasmid contigs, Table S5: Chilean isolates mobile elements.
