# Supplementary material for: Comparative Genomic Analysis of Novel Bifidobacterium longum subsp. longum Strains Reveals Functional Divergence in the Human Gut Microbiota
Source: Microorganisms. 2021 Sep 8;9(9):1906. doi: 10.3390/microorganisms9091906 (PMC8470182; doi:10.3390/microorganisms9091906)
Supplement: Supplementary file 1 [file microorganisms-09-01906-s001.zip › Supplementary Table S2.pdf]

Table S1: Virulence factor-associated genes found in Chilean isolates.

Isolate	Gene name	Associated VF	VFDB id	VF origin	Percent identity (%)
D4	tufA	EF-Tu	VF0460	Francisella tularensis	68.67
D4	wbtL	LPS	VF0542	Francisella tularensis	62.33
D4	wbtL	LPS	VF0542	Francisella tularensis	64.36
D4	wbtL	LPS	VF0542	Francisella tularensis	64.71
D4	ugd	Capsule	VF0560	Klebsiella pneumoniae	61.59
D4	ugd	Capsule	VF0560	Klebsiella pneumoniae	62.68
D4	flhA	Flagella	VF0394	Yersinia enterocolitica	85.42
D4	flhB	Flagella	VF0394	Yersinia enterocolitica	72.00
D4	flhB	Flagella	VF0430	Burkholderia pseudomallei	63.83
D4	yagV/ecpE	ECP	VF0404	Escherichia coli (EHEC)	96.12
E1	tufA	EF-Tu	VF0460	Francisella tularensis	68.67
M12	tufA	EF-Tu	VF0460	Francisella tularensis	68.67
M12	ugd	Capsule	VF0560	Klebsiella pneumoniae	61.59
S3	tufA	EF-Tu	VF0460	Francisella tularensis	68.67
S3	wbtL	LPS	VF0542	Francisella tularensis	64.36
S3	ugd	Capsule	VF0560	Klebsiella pneumoniae	62.68

Table S2: Antibiotic resistance genes found in Chilean isolates.

Isolate	Gene	Drug Class	AMR Gene Family	Percent identity (%)
D4	rpoB	rifamycin antibiotic	rifamycin-resistant beta-subunit of RNA polymerase (rpoB)	92.57
E1	ErmX	macrolide antibiotic	Erm 23S ribosomal RNA methyltransferase	90.14
E1	ErmX	lincosamide antibiotic	Erm 23S ribosomal RNA methyltransferase	90.14
E1	ErmX	streptogramin antibiotic	Erm 23S ribosomal RNA methyltransferase	90.14
E1	rpoB	rifamycin antibiotic	rifamycin-resistant beta-subunit of RNA polymerase (rpoB)	92.57
M12	rpoB	rifamycin antibiotic	rifamycin-resistant beta-subunit of RNA polymerase (rpoB)	92.57
S3	rpoB	rifamycin antibiotic	rifamycin-resistant beta-subunit of RNA polymerase (rpoB)	92.57
