# Supplementary figures and images for: Comparative Genomic Analysis of Novel Bifidobacterium longum subsp. longum Strains Reveals Functional Divergence in the Human Gut Microbiota
Source: Microorganisms. 2021 Sep 8;9(9):1906. doi: 10.3390/microorganisms9091906 (PMC8470182; doi:10.3390/microorganisms9091906)

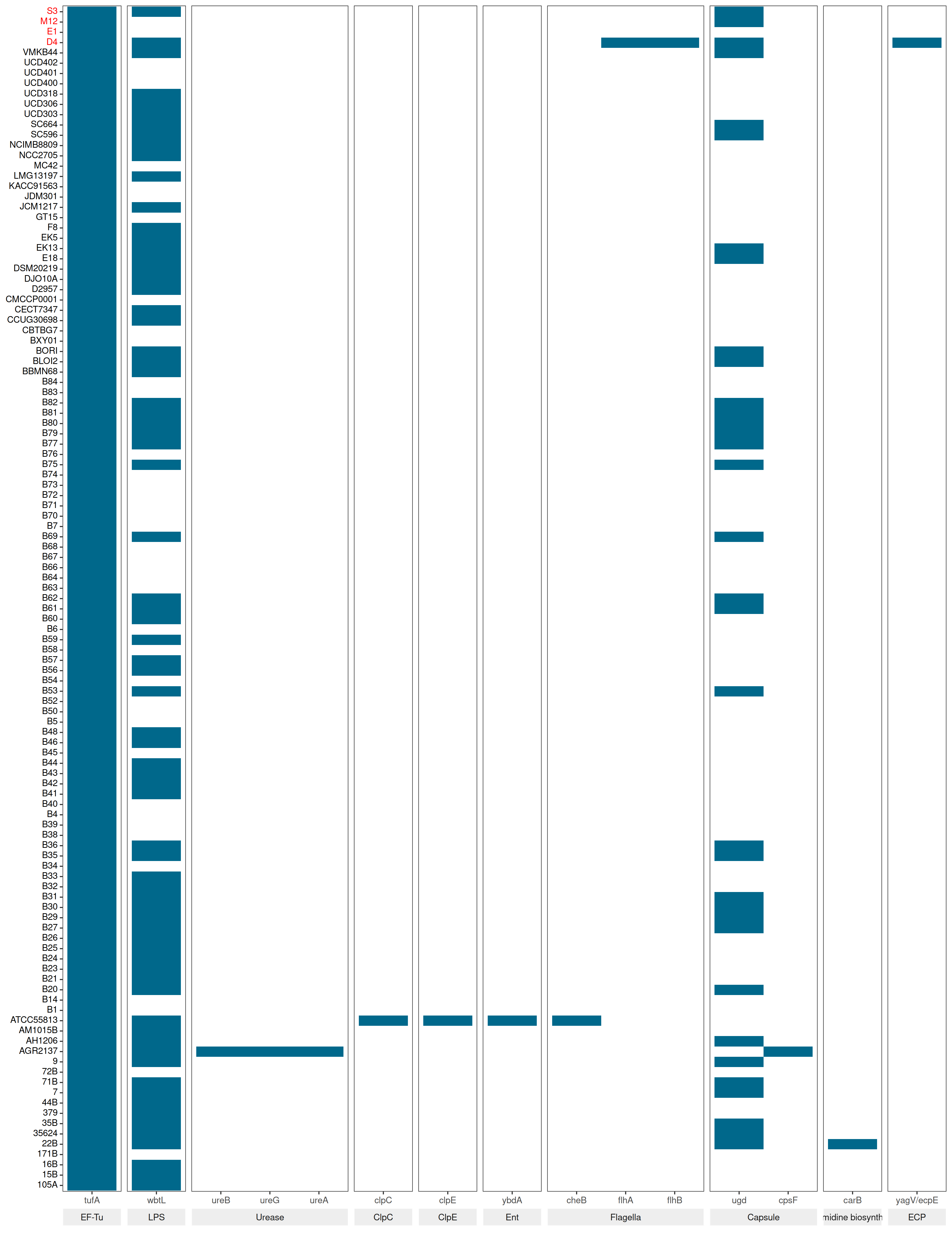

Supplement: Supplementary file 1 [file microorganisms-09-01906-s001.zip › Supplementary Figure S1.tiff]

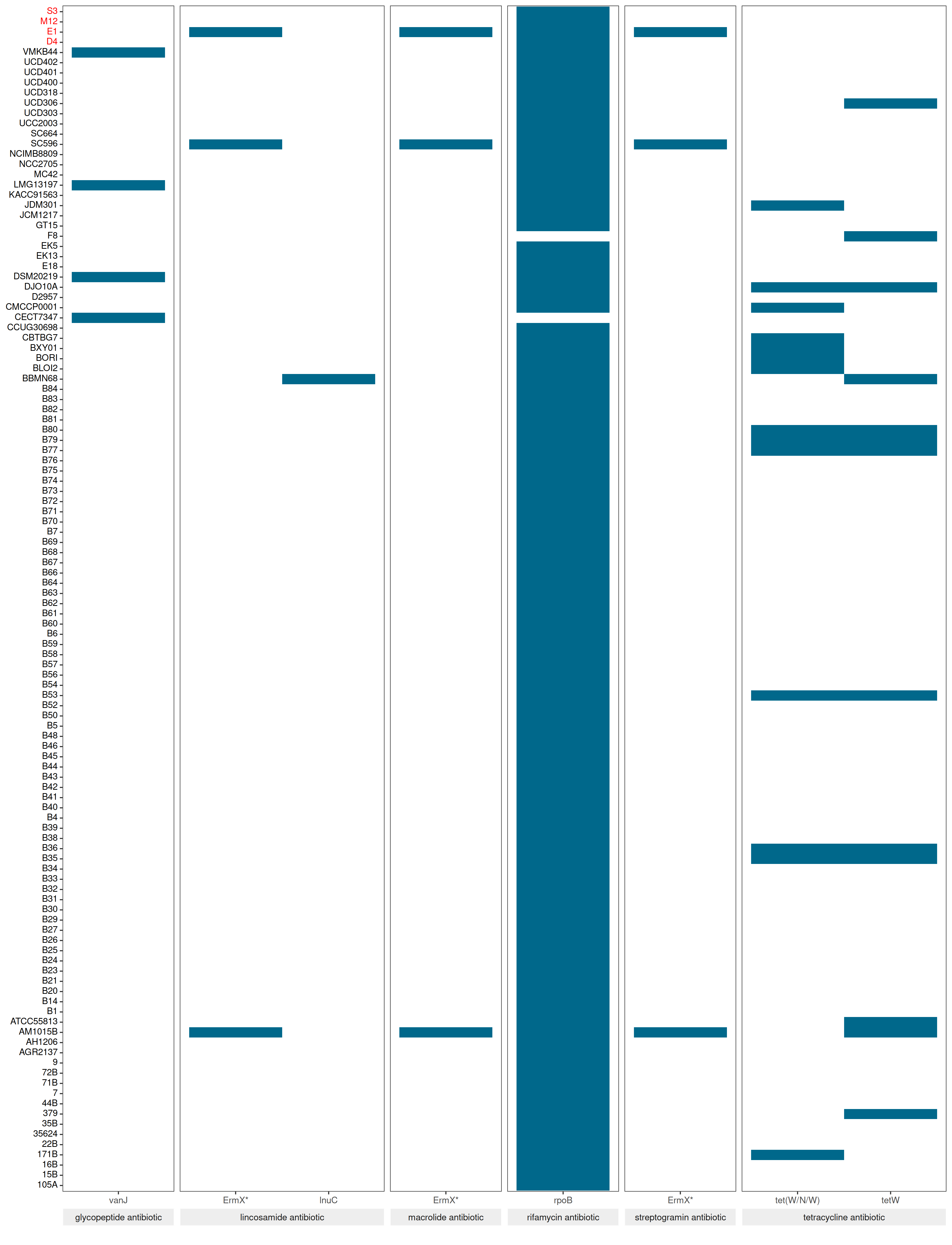

Supplement: Supplementary file 1 [file microorganisms-09-01906-s001.zip › Supplementary Figure S2.tiff]
